# Structure and comparative analysis of the mitochondrial genomes of *Liolaemus* lizards with different modes of reproduction and ploidy levels

**DOI:** 10.7717/peerj.10677

**Published:** 2021-03-22

**Authors:** Julian Valdes, Sergio Sebastian Samoluk, Cristian Simón Abdala, Diego Baldo, Guillermo Seijo

**Affiliations:** 1 Instituto de Botánica del Nordeste (UNNE-CONICET), Corrientes Capital, Corrientes, Argentina; 2 Unidad ejecutora Lillo (CONICET), Facultad de Ciencias Naturales e Instituto Miguel Lillo (IML), Universidad Nacional de Tucumán, San Miguel de Tucumán, Tucumán, Argentina; 3 Laboratorio de Genética Evolutiva, Instituto de Biología Subtropical (CONICET-UNaM), Facultad de Ciencias Exactas Químicas y Naturales, Universidad Nacional de Misiones, Posadas, Misiones, Argentina; 4 Facultad de Ciencias Exactas y Naturales y Agrimensura, FaCENA-UNNE, Corrientes Capital, Corrientes, Argentina

**Keywords:** Mitochondrial genomes, Lizards, Liolaemus species, Tandem repeats

## Abstract

*Liolaemus* is the most specious genus of the Squamata lizards in South America, presenting exceptional evolutionary radiation and speciation patterns. This recent diversification complicates the formal taxonomic treatment and the phylogenetic analyses of this group, causing relationships among species to remain controversial. Here we used Next-Generation Sequencing to do a comparative analysis of the structure and organization of the complete mitochondrial genomes of three differently related species of *Liolaemus* and with different reproductive strategies and ploidy levels. The annotated mitochondrial genomes of ca. 17 kb are the first for the Liolaemidae family. Despite the high levels of sequence similarity among the three mitochondrial genomes over most of their lengths, the comparative analyses revealed variations at the stop codons of the protein coding genes and the structure of the tRNAs among species. The presence of a non-canonical dihydrouridine loop is a novelty for the pleurodonts iguanians. But the highest level of variability was observed in two repetitive sequences of the control region, which were responsible for most of the length heterogeneity of the mitochondrial genomes. These tandem repeats may be useful markers to analyze relationships of closely related species of *Liolaemus* and related genera and to conduct population and phylogenetic studies.

## Introduction

The most representative Squamata in South America are members of the family Liolaemidae (Frost et al., 2001; Townsend et al., 2011; Pyron, Burbrink & Wiens, 2013; Abdala & Quinteros, 2014; Abdala et al., 2020). This family is arranged into three lineages, taxonomically treated as the *Ctenoblepharys*, *Liolaemus*, and *Phymaturus* genera (Abdala & Quinteros, 2014; Abdala et al., 2020; Villegas Paredes et al., 2020). *Liolaemus* is the most specious and diverse genus in South America with 283 species formally described (Abdala, Laspiur & Langstroth, 2020) with an average of 6.5 species described each year from 2008 to date (Abdala et al., 2020). This group has one of the broadest latitudinal and altitudinal distributions of any lizard genus and occupies mostly arid and semi-arid habitats in southern South America (Etheridge, 1995; Lobo, Espinoza & Quinteros, 2010). They cover a large range of environments that extends from Tierra del Fuego (its southernmost distribution) to Peru (its northernmost distribution), also inhabiting various regions in Argentina, Bolivia, Chile, Paraguay and the coasts of Brazil and Uruguay (Abdala & Quinteros, 2014) from the Atlantic and Pacific seashores to above the snow line over 5,000 m.a.s.l (Aparicio & Ocampo, 2010). Moreover, *Liolaemus* presents diverse morphological and ethological adaptations to micro-habitats, such as trees, rocks, open grasslands, sandy substrates and bush areas, among others (Pincheira-Donoso & Scolaro, 2007; Pincheira-Donoso, Harvey & Ruta, 2015). As a result, these lizards have acquired an extraordinary variability in sizes (from 50 mm to over 115 mm), body shapes, coloring patterns, and life histories, such as viviparity and oviparity (Abdala & Quinteros, 2014).

Due to the high morphological variability and wide distribution, many authors have divided and classified the species of this genus in different ways, describing other genera, subgenera, groups and species complexes that, in turn, have changed over time (Morando et al., 2004; Lobo, Espinoza & Quinteros, 2010; Abdala & Quinteros, 2014). The uncertainty to address a stable taxonomic treatment of *Liolaemus* has been associated with the fact that the group has radiated rapidly across South America (Grummer et al., 2018; Esquerré et al., 2019). As in most cases, these short time intervals between speciation events caused discordant divergence in morphology and molecular markers, and among loci obtained from different sources as well (Olave et al., 2014; Leaché et al., 2015). Thus, in addition to the complicate formal taxonomic treatment of these taxa, the recent radiation appears to be affecting phylogenetic analyses causing relationships among species to remain controversial (see Abdala, 2007; Morando et al., 2020).

Pioneer attempts to establish the relationships between the species of the *Liolaemus* were made using morphological characters (Laurent, 1983, 1985; Cei, 1986; Etheridge, 1995), isoenzymes (Young-Downey, 1998) and few nuclear and mitochondrial sequences (Schulte et al., 2000; Morando et al., 2004; Abdala, 2007). More recently, sequencing DNA markers associated with the restriction site (RAD-Seq) (Villamil et al., 2019) and few hundreds of nuclear loci with directed sequence capture (Morando et al., 2020) were also implemented to analyze the genus. However, despite the tendency to expand genome coverage, these approaches are based on partial regions of the genome, and most studies used markers developed based on nucleotide variability detected in other groups of reptiles. Moreover, the inferences of species relationships in *Liolaemus* using different markers hampered comparative and integrative studies.

Studies of sequence variability, gene disposition, and genetic codes of mitochondrial genomes provided a vast repertoire of phylogenetic markers, although it has not yet been fully explored in many animal groups (Bernt et al., 2013). Besides, although metazoan mitochondrial genomes are conserved in terms of structure, re-arrangements were recorded in many groups (Kumazawa et al., 1996; Macey et al., 1997; Mindell, Sorenson & Dimcheff, 1998; Rest et al., 2003) more conspicuously reported among parthenogenic lineages of lizards (Moritz & Brown, 1987; Moritz, 1991; Zevering et al., 1991; Fujita, Boore & Moritz, 2007). However, complete assembled mitogenomes are required within each group of organisms to detect, aside from genome-wide sequence variability, structural markers potentially useful for phylogenetic analyses.

In our work, the first mitcochondrial genomes of *Liolaemus* were assembled using Next-Generation Sequencing Technology to do a comparative analysis of the structure and organization of the complete mitogenomes of three differently related species of *Liolaemus* and with different reproductive strategies and ploidy levels. The species were selected from different phylogenetic clades, that is, *L. darwinii* and *L. parthenos* belong to the *L. darwinii* clade while *L. millcayac* belongs to the more basal clade *L. anomalus*. Moreover, *L. darwinii* and *L. millcayac* are sexually reproductive species, while *L. parthenos* is the only parthenogenetic triploid species known for pleurodont iguanian lizards, and the studies propose that it originated from hybridization between *L. darwinii* and other unknown species of the genus (Abdala et al., 2016). These are the first annotated mitogenomes for the Liolemidae family, and the comparative analyses revealed a particular structural characteristic of the *Liolaemus* mitogenomes among pleurodont iguanians, and hypervariable regions that can be adopted to improve further population or phylogenetic studies in *Liolaemus* and related genera.

## Materials and Methods

### Sample, DNA isolation and whole-genome sequencing

This study was conducted in accordance with international standards on animal welfare, as well as being compliant with national regulations and the “Comité Nacional de Ética en la Ciencia y la Tecnología” of Argentina. Specimens were euthanized with a 1% Alital solution, fixed with 10% formaldehyde and stored in 70% alcohol. Voucher of all the specimens are housed in the herpetological collections of the Fundación Miguel Lillo (FML, Tucumán, Argentina). All the collections were done under the permits 090/02, 034/06, 1397/07, 821/10, 296/10, 1259/10, 1296/10 issued by Departamento de Fauna, Mendoza, Argentina. For molecular studies, DNA was extracted from liver or muscle tissues stored in a freezer with 96% ethanol.

For genome sequencing, the genomic DNA of three *Liolaemus* species (*L. darwinii*, *L. parthenos* and *L. millcayac*) were isolated from ethanol-preserved liver samples using salt extraction protocol as outlined in Aljanabi (1997). The integrity and quality of each genomic DNA were checked by electrophoresis and spectrophotometry, respectively. The samples were remitted to Macrogen Inc., Korea, for library construction and sequencing. Briefly, DNA samples were randomly fragmented with Covaris and the libraries were then prepared using the Illumina TruSeq Nano DNA Kit (550 bp insert size). These libraries were sequenced on Illumina NovaSeq platform to obtain 2 × 150 bp paired-end reads.

### Assembly and annotation

All the bioinformatic analysis was carried out using the computational infrastructure of the Instituto de Botánica del Nordeste (Universidad Nacional del Nordeste—CONICET). After removing low quality sequences (Phred scores <30) and trimming Illumina adapters with Trim Galore (https://www.bioinformatics.babraham.ac.uk/projects/trim_galore/), the de novo assemblies of mitochondrial genomes were performed using NOVOPlasty v2.6.3 (Dierckxsens, Mardulyn & Smits, 2017). This software assembles organelle genomes using a seed-and-extend algorithm from WGS data, starting from a related or distant single “seed” sequence and an optional “bait” reference mitogenome. For mitogenome assembly of *Liolaemus* species, we used the nucleotide sequence of the cytochrome oxidase subunit 1 and the mitogenome of *Anolis punctatus* as seed and bait reference mitogenome (NCBI RefSeq NC_044125.1), respectively. These assemblies were performed following the developer’s suggestions and using a kmer size of 23. The assembled mitogenomes of the three *Liolaemus* species were annotated using the software GeSeq (Tillich et al., 2017), selecting the options to perform tRNAscan-SE and BLAT using the sequence for *Anolis punctatus*. Then, annotations were manually checked to correct misannotated regions. The mitogenome maps were drawn using GenomeVx online tool (Conant & Wolfe, 2008). Complete annotated mitogenomes were deposited in the GenBank with the following accession numbers: MT810467 (*L. darwinii*), MT810468 (*L. millcayac*) and MT810469 (*L. parthenos*).

### Comparative analysis

The assembled *Liolaemus* mitogenomes were compared to calculate the nucleotide composition, Relative Synonymous Codon Usage (RSCU), non-synonymous (Ka) and synonymous (Ks) substitutions, gene re-arrangements, and variation sites.

Codon usage statistics were calculated using DnaSP version 5.0 (Librado & Rozas, 2009). The nucleotide sequences of each PCG were aligned based on the amino acid sequences in TranslatorX (Abascal, Zardoya & Telford, 2010) with MAFFT algorithm. The ratios of non-synonymous substitutions (Ka) and synonymous (Ks) substitutions were estimated in DnaSP version 5.0 (Librado & Rozas, 2009). To assess interspecific variation, pairwise comparisons among the three *Liolaemus* mitochondrial genomes were made with mVISTA program (Frazer et al., 2004) in Shuffle-LAGAN mode. The mitogenomes were aligned, and the overall sequence identity was plotted, using the annotation of *L. parthenos* as reference.

## Results

### Genome sequencing and assembly of mitogenomes

A summary of the sequencing and mitogenome assembly outputs is shown in the Table S1. Briefly, after quality filtering of the Illumina NovaSeq raw data, a total of 42,135,996 reads were obtained for *Liolaemus parthenos*, 39,057,114 reads for *L. darwinii* and 44,253,406 reads for *L. millcayac*. These sequence data were used as input to assemble the mitogenome in each species. The assembled mitogenomes of *Liolaemus parthenos*, *L. darwinii*, and *L. millcayac* consisted in circular molecules of 16,838 bp, 16,974 bp and 16,945 bp, respectively. These assemblies were highly covered by sequence reads (average coverage of 1,171 in *L. parthenos*, 715 in *L. darwinii* and 572 in *L. millcayac*) giving the chance to develop robust mitogenomes.

### Genome organization and composition

**Figure 1:**
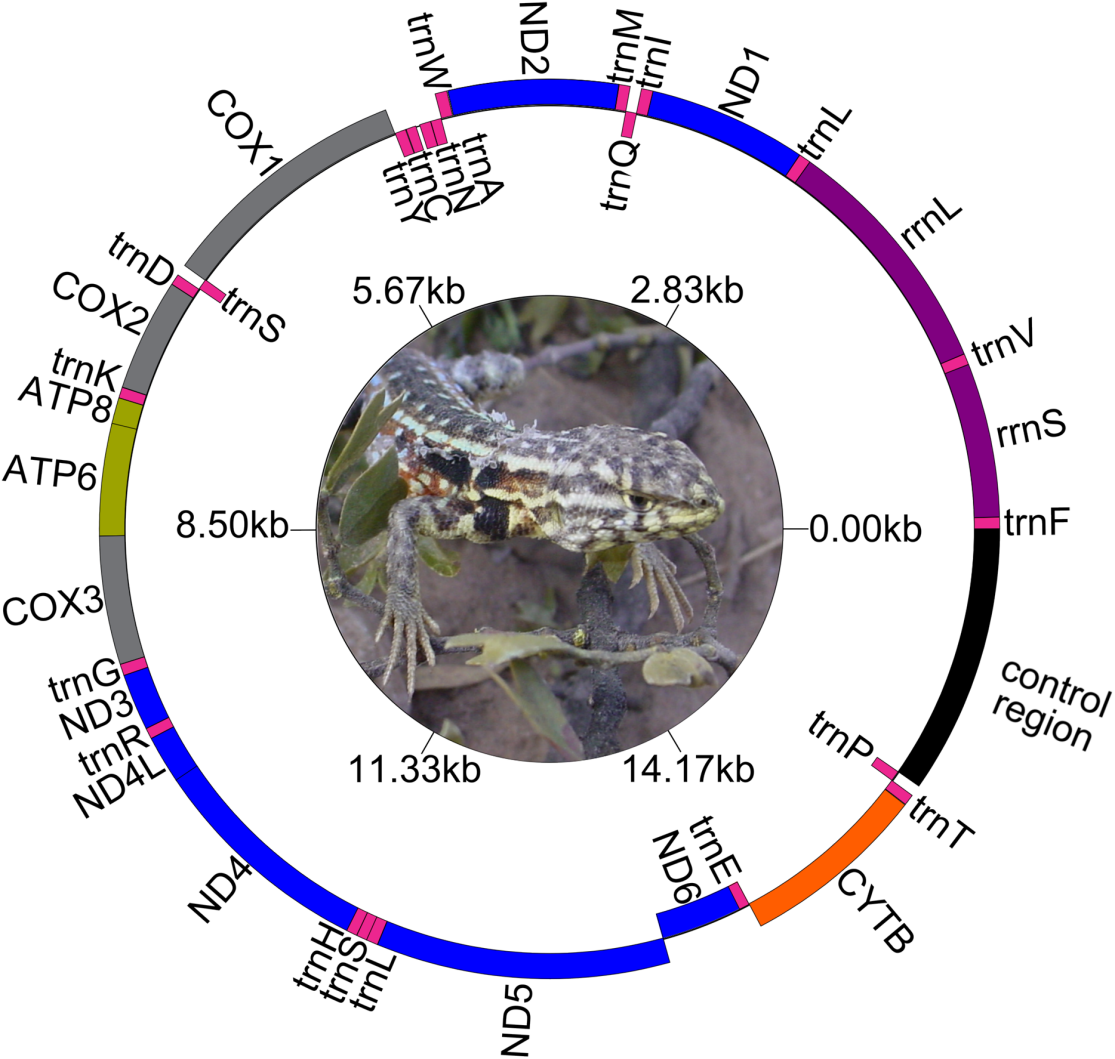
Map of the complete mitogenome of *Liolaemus darwinii*. Ph: Cristian Abdala.

**Figure 2:**
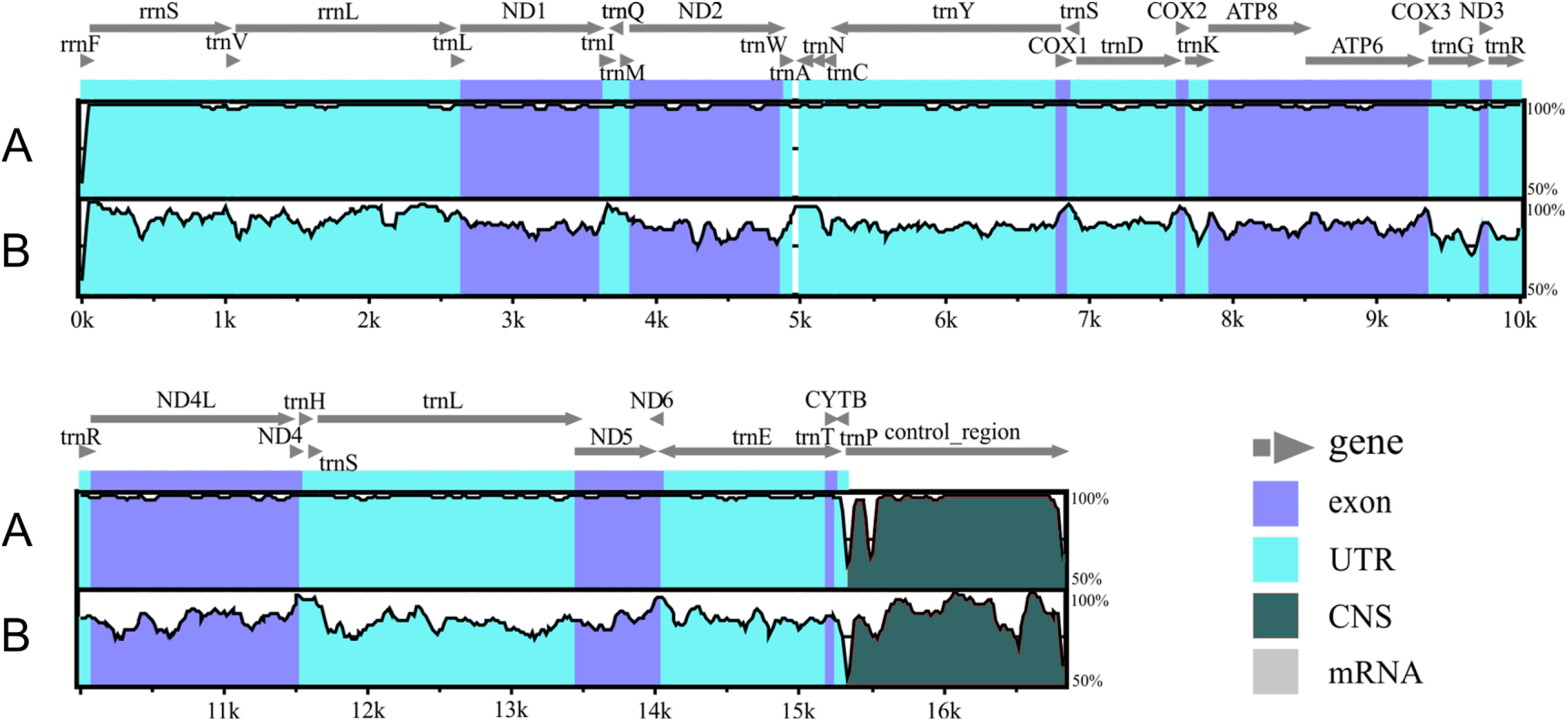
Alignment of the *Liolaemus* species mitogenomes. The sequence of *L. parthenos* mitogenome was compared to those of *L. darwinii* (A) and *L. millcayac* (B) using mVISTA for the alingment. Grey arrows above the alignment indicate genes and their orientation.

The genetic maps of *Liolaemus* mitogenomes are shown in Fig. 1. They were composed of 37 genes (13 PCGs, 22 tRNAs and two rRNAs) and two non-coding regions (the origin for light-strand replication and a control region). The mitochondrial genomes showed identical gene order and organization (Fig. 2). In addition, the alignment revealed high sequence similarity across most of the extension of the three mitogenomes. The sequence identities in pairwise comparisions were 99,44% (*L. parthenos*–*L. darwinii*), 86,46% (*L. millcayac*–*L. parthenos*) and 86,40% (*L. millcayac*–*L. darwinii*). The overall base composition of the three mitogenomes was very similar, showing a bias towards A and T nucleotides (ca. 61% Table 1).

**Table 1:** Nucleotide composition of the different regions of the *Liolaemus* mitogenomes.

	% A	% T	% C	% G
**Whole genome**				
*L. parthenos*	33.7	27.6	24.8	13.9
*L. darwinii*	33.6	27.6	24.9	13.9
*L. millcayac*	33.7	27.0	25.2	14.1
**PCGs**				
*L. parthenos*	31.4	30.0	24.9	13.6
*L. darwinii*	31.5	30.0	24.9	13.6
*L. millcayac*	31.5	29.3	25.5	13.7
**tRNAs**				
*L. parthenos*	31.4	27.6	19.4	21.6
*L. darwinii*	31.4	27.6	19.4	21.6
*L. millcayac*	31.0	28.2	19.0	21.8
**rRNAs**				
*L. parthenos*	37.8	21.7	22.6	17.9
*L. darwinii*	37.8	21.7	22.6	17.9
*L. millcayac*	37.6	21.6	22.7	18.1
**CR**				
*L. parthenos*	34.6	31.6	20.8	13.0
*L. darwinii*	32.6	31.4	21.9	14.0
*L. millcayac*	34.8	30.0	21.0	14.1

Protein-Coding Genes were organized in a large cluster on the heavy strand, except for the PCG “ND6” and eight tRNAs (tRNAGln, tRNAAla, tRNAAsn, tRNACys, tRNATyr, tRNASer (UCN), tRNAGlu and tRNAPro) which were organized on the light strand. Intergenic spacers were observed both on the H and the L strands. Four intergenic spacers (ND2- tRNATrp, COX2-tRNALys, tRNALeu-ND5 and CYTB-tRNAThr) were observed on the H- strand and four (tRNAAla-tRNAAsn, tRNAAsn-tRNACys, tRNACys-tRNATyr and ND6- tRNAGlu) on the L- strand. Overlapped segments were observed on ATP8-ATP6, ATP6-COX3, ND4L-ND4, tRNASer-tRNALeu, all in the H-strand. However, in *L. millcayac*, overlapped segments were observed on COX2-tRNALys and tRNALeu-ND5 and one intergenic spacer was found between the genes ATP6 and COX3. The sizes of spacers and overlaps were variable in the three species analyzed were inferred from annotations of Table 2, and their evolutionary significance should be tested in a broader context.

**Table 2:** Organization of the mitogenomes in *Liolaemus* species. DAR*: L. darwinii*, PAR: *L. parthenos*, MIL: *L. millcayak*.

Gene	Strand			Location			Size (bp)			Anticodon	Start codon/stop codon		
	DAR	PAR	MIL	DAR	PAR	MIL	DAR	PAR	MIL	All spp.	DAR	PAR	MIL
trnF tRNA	+	+	+	1–71	1–71	1–71	71	71	71	GAA			
rrnS rRNA	+	+	+	72–1,011	72–1,011	72–1,009	940	940	938				
trnV tRNA	+	+	+	1,012–1,079	1,012–1,079	1,010–1,080	68	68	71	TAC			
rrnL rRNA	+	+	+	1,080–2,577	1,080–2,575	1,081–2,572	1,498	1,496	1,492				
trnL tRNA	+	+	+	2,578–2,651	2,576–2,649	2,573–2,646	74	74	74	TAA			
ND1	+	+	+	2,652–3,617	2,650–3,615	2,647–3,612	966	966	966		ATG/TAG	ATG/TAG	ATG/TAG
trnI tRNA	+	+	+	3,617–3,687	3,615–3,685	3,612–3,682	71	71	71	GAT			
trnQ tRNA	–	–	–	3,686–3,756	3,685–3,754	3,682–3,751	71	71	71	TTG			
trnM tRNA	+	+	+	3,756–3,824	3,754–3,822	3,751–3,820	69	69	70	CAT			
ND2	+	+	+	3,825–4,862	3,823–4,860	3,821–4,858	1,038	1,038	1,038		ATG/TAA	ATG/TAA	ATG/TAG
trnWtRNA	+	+	+	4,866–4,938	4,864–4,936	4,857–4,926	72	72	70	TCA			
trnA tRNA	–	–	–	4,941–5,009	4,939–5,007	4,929–4,996	69	69	67	TGC			
trnN tRNA	–	–	–	5,011–5,083	5,009–5,081	4,998–5,070	73	73	73	GTT			
trnC tRNA	–	–	–	5,109–5,174	5,107–5,172	5,096–5,160	68	68	64	GCA			
trnY tRNA	–	–	–	5,178–5,248	5,176–5,246	5,170–5,240	71	76	70	GTA			
COX1	+	+	+	5,250–6,797	5,248–6,795	5,242–6,789	1,548	1,548	1,548		GTG/AGA	GTG/AGA	GTG/AGA
trnS tRNA	–	–	–	6,793–6,862	6,791–6,860	6,785–6,854	70	70	70	TGA			
trnD tRNA	+	+	+	6,866–6,933	6,864–6,931	6,858–6,925	68	68	68	GTC			
COX2	+	+	+	6,934–7,620	6,932–7,618	6,926–7,612	687	687	687		ATG/TAA	ATG/TAA	ATG/TAA
trnK tRNA	+	+	+	7,622–7,688	7,620–7,686	7,614–7,680	67	67	67	TTT			
ATP8	+	+	+	7,689–7,856	7,687–7,854	7,681–7,848	168	168	168		ATG/TAA	ATG/TAA	ATG/TAA
ATP6	+	+	+	7,847–8,530	7,845–8,528	7,839–8,522	684	684	684		ATG/TAA	ATG/TAA	ATG/TAA
COX3	+	+	+	8,530–9,313	8,528–9,311	8,525–9,308	784	784	784		ATG/*T	ATG/*T	ATG/*T
trnG tRNA	+	+	+	9,314–9,383	9,312–9,381	9,309–9,378	70	70	70	TCC			
ND3	+	+	+	9,384–9,729	9,382–9,727	9,379–9,724	346	346	346		ATG/*T	ATG/*T	ATG/*T
trnR tRNA	+	+	+	9,730–9,796	9,728–9,794	9,725–9,791	66	66	67	TCG			
ND4L	+	+	+	9,797–10,093	9,795–10,091	9,792–10,088	297	297	297		ATG/TAA	ATG/TAA	ATG/TAA
ND4	+	+	+	10,087–11,467	10,085–11,465	10,082–11,462	1,381	1,381	1,381		ATG/*T	ATG/*T	ATG/*T
trnH tRNA	+	+	+	11,468–11,534	11,466–11,532	11,463–11,529	67	67	67	GTG			
trnS tRNA	+	+	+	11,535–11,600	11,533–11,598	11,530–11,595	66	66	66	GCT			
trnL tRNA	+	+	+	11,600–11,670	11,598–11,668	11,595–11,665	71	71	71	TAG			
ND5	+	+	+	11,672–13,462	11,670–13,460	11,667–13,457	1,791	1,791	1,791		ATG/TAA	ATG/TAA	ATG/TAA
ND6	–	–	–	13,458–13,982	13,456–13,980	13,453–13,977	525	525	525		ATG/AGA	ATG/AGA	ATG/AGG
trnE tRNA	–	–	–	13,984–14,052	13,982–14,050	13,978–14,046	68	68	67	TTC			
CYTB	+	+	+	14,051–15,190	14,049–15,188	14,045–15,184	1,140	1,140	1,140		ATG/TAA	ATG/TAA	ATG/TAG
trnT tRNA	+	+	+	15,194–15,261	15,192–15,259	15,184–15,251	68	68	68	TGT			
trnP tRNA	–	–	–	15,262–15,329	15,260–15,327	15,252–15,319	68	68	68	TGG			
C R	+	+	+	15,330–16,974	15,328–16,838	15,320–16,945	1,645	1,511	1,626				

### Protein coding genes

**Figure 3:**
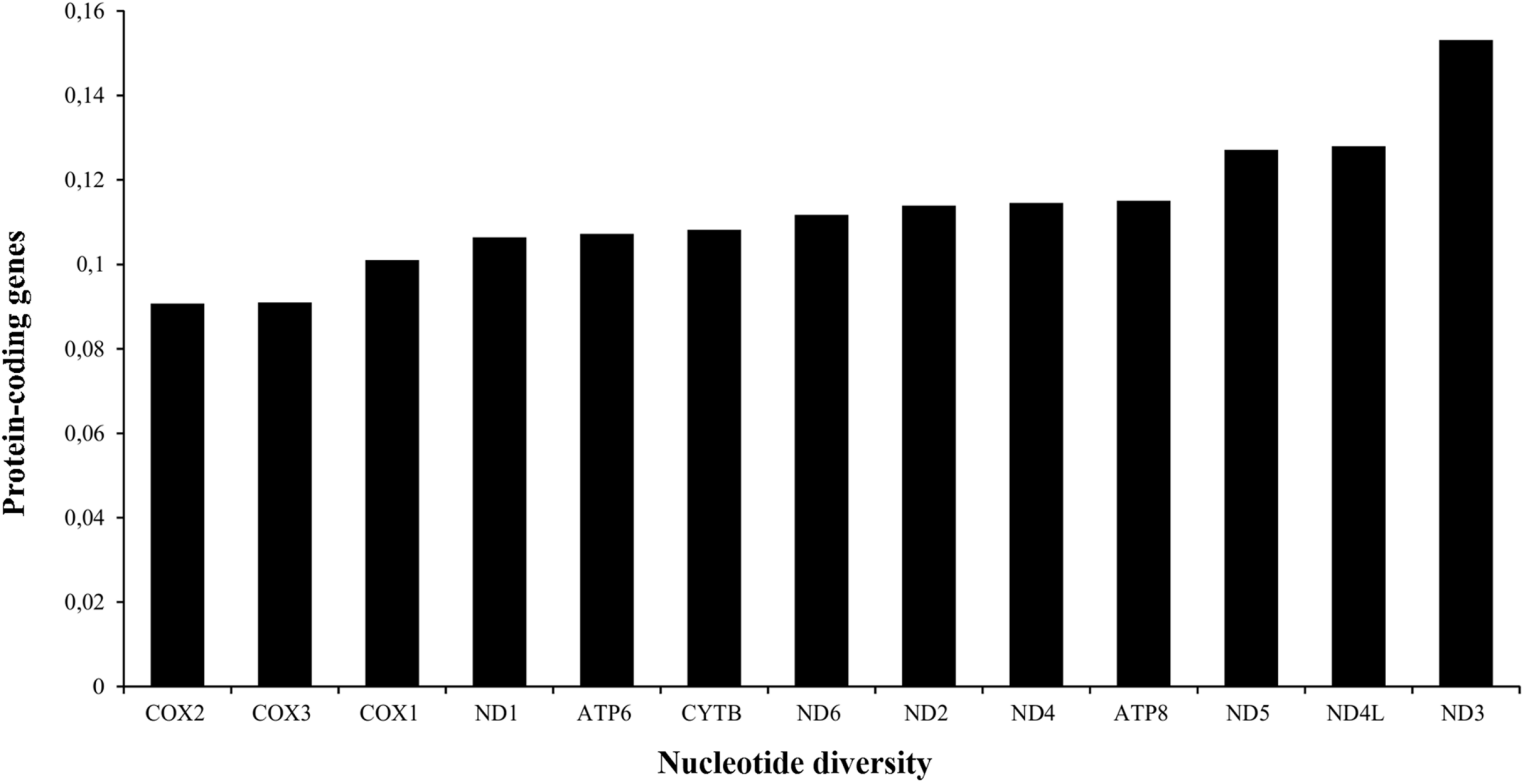
Nucleotide diversity of the PCGs in the mitogenomes of *Liolaemus* species.

The A+T content of PCGs was about 61% in the three species analyzed (Table 1). The total length of the 13 PCGs was conserved in *Liolaemus* species (11,355 bp). This value accounted for approximately 67% of the total mitochondrial genome length. The sizes of the different protein-coding genes were also conserved in the three species analyzed (Table 2), being the NAD5 the longest (1,791 bp) and ATP8 the shortest one (168 bp). The analysis of the nucleotide diversity revealed high conservation of nucleotide sequences of the 13 PCGs. Among them, the most conserved genes were COX2 (0.0907) and COX3 (0.0909), whereas ND3 (0.1532) was the less conserved PCG (Fig. 3).

Most of the PCGs showed the same start codon in the three species (Table 2). Twelve genes started with ATG, while only the COX1 started with GTG. The four stop codons of the vertebrate mitochondrial code were observed in the species here analyzed. The sequence “TAA” was the most frequent and it was present in COX2, ATP8, ATP6, ND4L and ND5 in the three species, and also in the genes ND2 and CYTB in the species *L. darwinii* and *L. parthenos*. The stop codon “AGA” was observed in the ND6 (only in *L. darwinii* and *L. parthenos*) and COX1 genes (all species). The sequence “TAG” was only found in the gene ND1 for *L. darwinii* and *L. parthenos*, and in the genes ND1, ND2 and CYTB for *L. millcayac*. The stop codon “AGG” was only present in the gene ND6 of *L. millcayac*. Three genes (COX3, ND3 and ND4) showed incomplete stop codons “T”, which is completed by the addition of 3′ “A” residues to the mRNA. These results showed that the different *Liolaemus* species present variability in the stop codons of some genes whose usefulness as diagnostic characters should be rigorously sampled in the genus.

**Figure 4:**
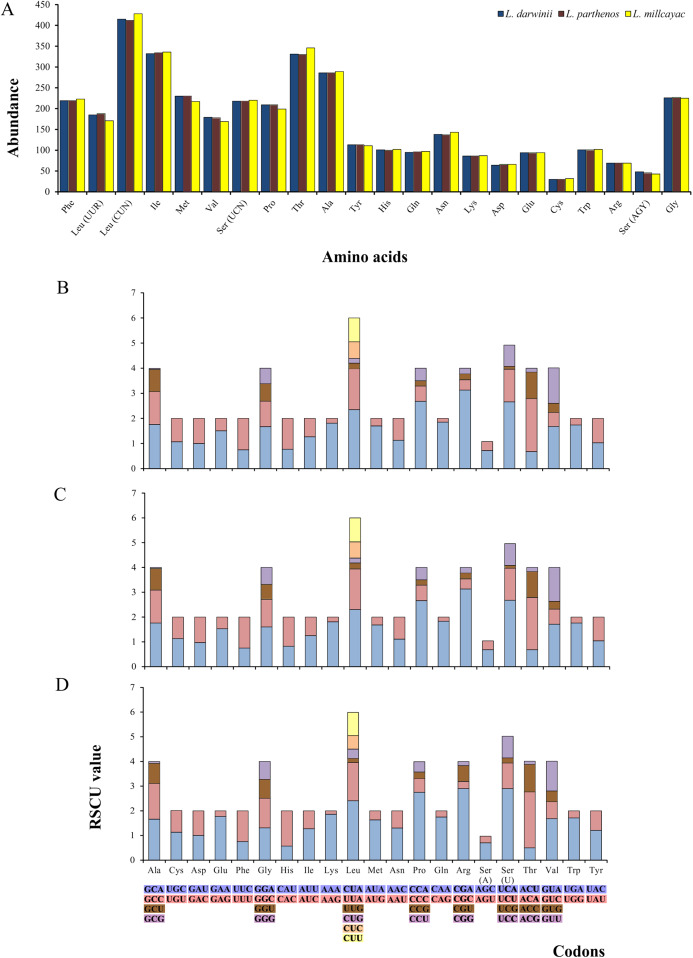
(A) Abundance of codon families. Relative Synonymous Codon Usage (RSCU) in the 13 PCGs in the mitogenomes of *Liolaemus darwinii*, (B) *L. parthenos* (C) and *L. millcayac* (D).

**Figure 5:**
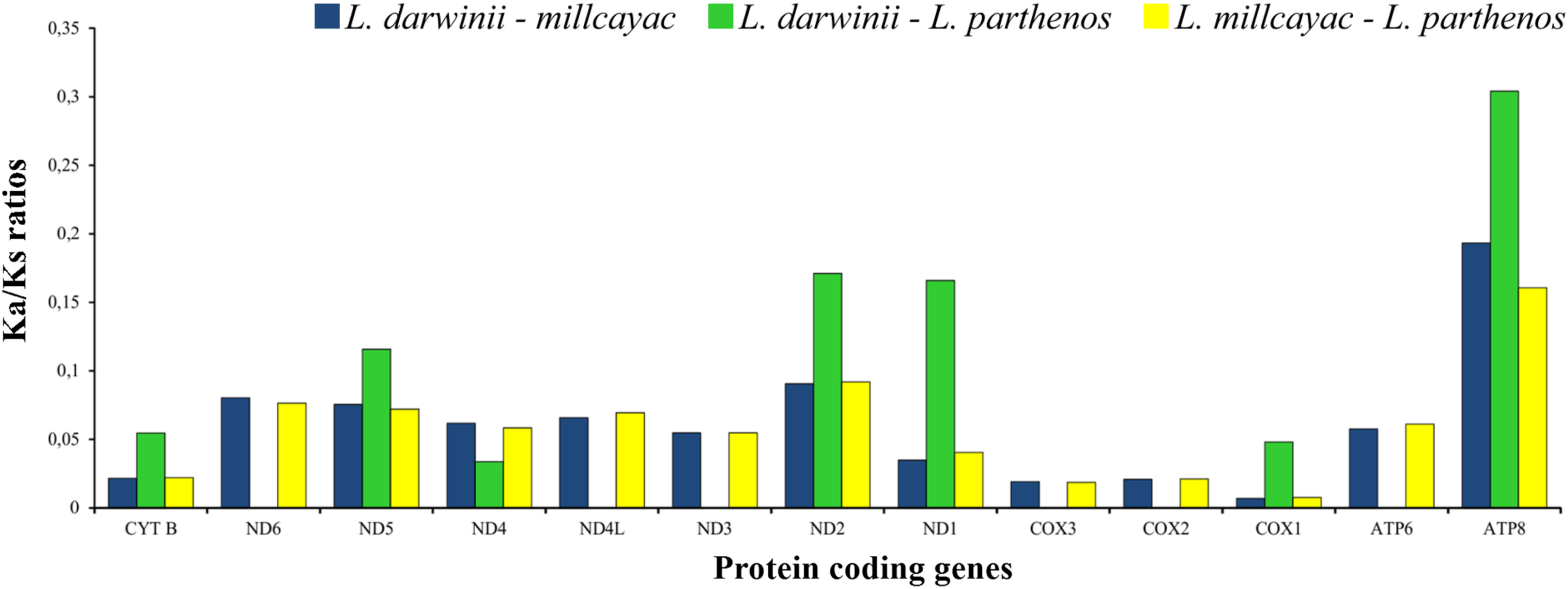
The ratios of non-synonymous (Ka) versus synonymous (Ks) substitutions (Ka/Ks ratios) of the 13 PCGs in the mitogenomes of *Liolaemus* especies.

The abundance of codon families and Relative Synonymous Codon Usage (RSCU) in the 13 PCGs in *Liolaemus* mtDNAs are shown in Fig. 4A. A total of 3,774 non-stop codons with a very similar behavior were found in each species. The most frequently used amino acids were Leucine (Leu), followed by Isoleucine (Ile), Threonine (Thr) and Alanine (Ala) (Fig. 4A). The analysis of relative synonymous codon usage (RSCU) revealed the presence of 60 codons representing 22 amino acids. Each amino acid was represented by two to four codons (Figs. 4B–4D), being CTA (Leu), ATT (Ile), TAC (Thr) and AAC (Ala), the most frequently used for the three species. The ratios of non-synonymous (Ka) versus synonymous (Ks) substitutions (Ka/Ks ratios) of the 13 PCGs were less than 1; among which ATP8 and COX1 had the highest and lowest rates, respectively (Fig. 5). This relationship suggests that a strong purifying and negative selection may be operating on these genes.

### Ribosomal and transfer RNAs

The two rRNA genes (rrnS and rrnL) are typically between tRNAPhe and tRNALeu (UUA) and separated by tRNAVal (Fig. 1). Both rRNAs were AT-rich (ca. 59.5%), and the length of each rRNAs was identical in the three species (Table 1). The level of sequence identity of rRNA genes followed the general findings for the whole mitogenome, being highest between *L. darwinii* and *L. parthenos* (Table 2).

The 22 tRNA genes were interspersed between the rRNAs and the protein-coding region (Fig. 1). The nucleotide composition of these tRNAs showed approximately 59% of A and T (Table 1) and their sequence lengths were very similar in the three species except for tRNATyr, which showed a size variation of 6 bp (Table 2). The sequences of all tRNA genes displayed the typical cloverleaf secondary structure composed of four domains and a short variable loop: the acceptor stem, the dihydrouridine stem and loop (DHU), the anticodon stem and loop, the thymidine stem and loop (TψC), and the variable (V) loop (Figs. S1–S3) 1. However, some variants in the secondary structure were observed among species in the acceptor, DHU, TψC and anticodon domains of the different tRNAs. A deletion of stem, loop and stem and loop of the DHU domain were observed in the trnI, trnC and trnS genes, respectively. Mismatched base pairs in the acceptor arm (trnF, trnQ, trnM, trnD, trnK, trnR, trnH and trnT), DHU arm (trnW) and TψC arm (trnM, trnW and trnS) were observed in all the species, but a mismatched pair in the anticodon arm of trnP was only present in *Liolaemus millcayac*. Also, an unpaired single nucleotide was detected in TψC arm of trnK in the three species analyzed.

### Composition and structure of non- coding regions

**Figure 6:**
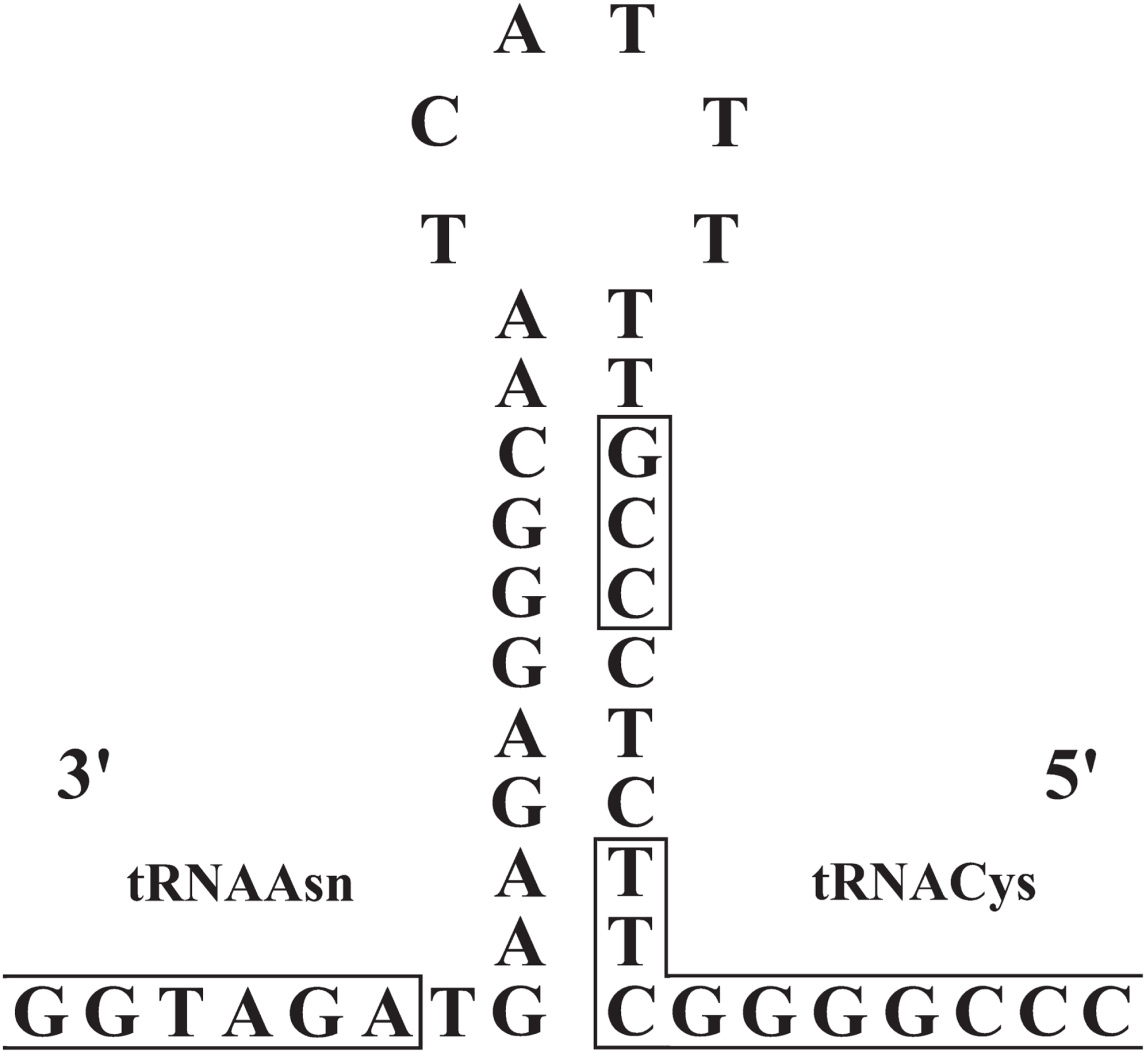
Stem-and-loop structures in the typical position for OL between the tRNAAsn and tRNAcys genes in the mitogenomes of *Liolaemus* species. The box in the stem denotes the potential 3′-GCC-5′ light-strand elongation start point (Macey, Schulte & Larson, 2000).

The origin of light-strand replication (OL) was located in the WANCY region (between the tRNAAsn and tRNACys genes) as expected for vertebrates. The OL stem and loop structures of the three *Liolaemus* species (Fig. 6) were similar to the consensus sequences in squamate lizards (Macey, Schulte & Larson, 2000). The stem region presented a potential 3′-GCC-5′ light-strand elongation start point as was described in mouse (Brennicke & Clayton, 1981) and later observed in lizards of the *Leiolepis* genus (Macey et al., 2006). In addition, the 3′-GBCCB-5′ sequence was found downstream of the stem region, which is a variant of the sequence 3′-GGCCG-5′ required for in vitro replication of human mitochondrial DNA (Hixson, Wong & Clayton, 1986).

**Figure 7:**
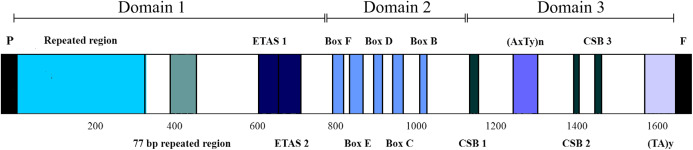
Features in control regions of *Liolaemus* lizards. The CR is divided into three domains: Domain 1 (TAS), Domain 2 (CD) and domain 3 (CSB). The two types of AT-rich sequences of the CRs are indicated as (AxTy)n and (TA)y. P: tRNAPro, F: tRNAPhe.

The CRs of *L. darwinii*, *L. parthenos* and *L. millcayac* were 1,645 bp, 1,511 bp and 1,626 bp in length, respectively (Table 2), as expected for the Iguanidae (Okajima & Kumazawa, 2010). The CRs showed a high content of A and T nucleotides (65% on average, Table 1) and localized in the same position reported for most vertebrates, between the tRNAPro and tRNAPhe genes (Boore, 1999). They are composed by three domains: TAS, CD and CSB (Fig. 7). The TAS domain (domain 1) was the most variable both in length (from 644 bp in *L. parthenos* to 755 bp in *L. millcayac*) and nucleotide diversity “π” (0.08712), while CD domain (domain 2) was the most conserved, both in sequence length (341 bp in the three species) and nucleotide diversity (0.02933). Intermediate values of sequence length (from 526 bp in *L. parthenos* to 530 bp in *L. millcayac*) and nucleotide diversity (0.06460) were observed in the CSB domain (domain 3). Domain 1 presented the ETAS (extended termination associated sequences) like sequences, the domain 2 had five conserved blocks (F, E, D, C and B) and the domain 3 contained three conserved blocks (CSB-1, 2 and 3). The arrangement observed for the control region is the most frequently reported for vertebrates (Satoh et al., 2016).

Aside from the conserved motifs, repeated regions were found in two domains of the mitochondrial CRs of *Liolaemus* species (Table 3). Two arrays of tandem repeats were localized in the domain 1. The first one was localized upstream of the ETAS-like sequences and consisted of repeat units of 33 bp (*L. darwinii* and *L. parthenos*) and 34 bp (*L. millcayac*). Importantly, the copy number of these repeat units was variable among the species, ranging from 5 (*L. millcayac*) to 9 (*L. darwinii*). This variation in copy number is the responsible of most of the length difference observed in the control region among the *Liolaemus* species. The second array detected in domain 1 was localized between the first tandem repeat region and ETAS-like sequences and, it was formed by two tandemly repeated units of 77 bp (*L. darwinii* and *L. parthenos*) and 76 bp (*L. millcayac*). In contrast with the first array, the copy number of the latter was conserved in the three species analyzed (two copies). Two types of AT-rich sequences, (AxTy)n and (TA)n, were found in the domain 3 of the CRs of the three *Liolaemus* species. The first one occurred between CSB-1 and CSB-2 motifs, while the second was localized 5′ to the tRNAPhe gene. The (AxTy)n sequence was composed of an 11 bp (AAATTAAATTA) unit repeated 5 times in *L. darwinii* and *L. parthenos*, while in *L. millcayac*, it was composed of a slightly different motif (AAATCAAATTA) repeated seven times. By contrast, the (TA)n sequence consists of a highly conserved motif of nearly 50 repetitions of TA repeats in the three species.

**Table 3:** Features of tandem repeats in control regions of the three analyzed *Liolaemus* species.

Species	Repeat unit (consensus)	Length (bp)	Copy no.	Domain	Nucleotide position
*L. darwinii*	ATACCTAGCCACCTCCGGGTGGCTTTATTGCCG	33	9	1	8–326
	CCCCACGAATAATAAGCAGGGAAAACAACCTACATTACTACACAATATACTATGTATATCGTGCATACACCTATTTT	77	2	1	387–553
	AAATTAAATTA	11	5	3	1,226–1,288
	TA	2	50	3	1,547–1,642
*L. parthenos*	AGCCACCTCCGGGTGGCTTTATTGCCGATAACT	33	5	1	14–194
	CCCCACGAATAATAAACAGGGAAAACAACCTACATTACTATATGATATTCTATGTATATCGTGCATACATTTCTTTT	77	2	1	255–409
	AAATTAAATTA	11	5	3	1,094–1,156
	TA	2	49	3	1,415–1,508
*L. millcayac*	AAAACCAGCCACCTCCGGGTGGCTTAGTTGCCGA	34	9	1	11–309
	CCCCATGAATAATAAGCAGGGAAAACCCATACATTACTATATGATATTCTATGTATATCGTGCATACATTTCTTTT	76	2	1	366–519
	AAATCAAATTA	11	7	3	1,195–1,275
	TA	2	51	3	1,525–1,623

## Discussion

The appearance of Next Generation Sequencing technologies together with the development of bioinformatic pipelines has facilitated the high-quality assembly of organelle genomes in eukaryote species. Here, through the analysis of Illumina paired-end reads, we provided the complete structure and organization of the mitochondrial genomes of three differently related species of *Liolaemus* with different reproductive strategy and ploidy levels, looking for variable regions useful for phylogenetic and population analysis.

The assembled sequences of the three *Liolaemus* mitogenomes are typical circular DNA molecules of similar size (approximately 16.9 kb). The content and order of genic and non-genic regions showed a high structural conservation among the species analyzed and, in general, when compared to other lizard genomes (Amer & Kumazawa, 2005; Okajima & Kumazawa, 2010; Nogueira Dumans et al., 2019). The low variability observed among the species analyzed is in accordance with the relatively recent radiation of the *Liolaemus boulengeri* group (Morando et al., 2020). Our results showed that the mitogenome of *L. parthenos* has not undergone any gene order re-arrangement during its evolutionary history as was previously recorded for several unisexual lizards (Moritz & Brown, 1987; Moritz, 1991; Zevering et al., 1991; Fujita, Boore & Moritz, 2007) suggesting structural conservation of the *Liolaemus* mitogenomes despite the mode of species reproduction and ploidy level.

Despite the high gene conservation at interspecific level, some structural variations were observed in the secondary structure of tRNA genes, particularly the genes trnS2 and trnC. The structural variation in the trnS genes has been reported in diverse lineages of vertebrates, such as Artiodactyla (Zhou et al., 2019), Galliformes (Li, Huang & Lei, 2015), Anura (Yang et al., 2018). By contrast, the phenomenon of the trnC gene lacking a canonical DHU arm is not common in the mitogenomes of vertebrates and was only found in acrodont lizards so far (Macey, Schulte & Larson, 2000). Thus, the finding of a non-canonical structure on DHU arm in *Liolaemus* constitutes the first report in the pleurodont iguanians. Moreover, the derived secondary structures of mitochondrial tRNAs found here have useful characters for interspecific phylogenetic studies in *Liolaemus*, since estimates of homoplastic changes for tRNA secondary structural characters were reported to be several (more than five) times less than for base position changes in lizards (Macey, Schulte & Larson, 2000).

The control regions were identified as those with the lowest values of sequence identity and are further analyzed below. In fact, the differences in the genome length were mainly due to the variable number of repeats in these regions. This degree of similarity in the pairwise comparison is in agreement with the phylogenetic relationships; whereas *L. millcayac* is placed in the early-diverged lineage *L. anomalus* clade; *L. darwinii* and *L. parthenos* are in the *L. darwinii* clade (Schulte et al., 2000; Morando et al., 2004; Abdala et al., 2016). Moreover, in their phylogenetic hypotheses with a few mitochondrial genes, Abdala et al. (2016) recovered *L. parthenos* nested within *L. darwinii* and proposed it as their maternal ancestor. The comparisons of the whole mitochondrial genomes are congruent with this hypothesis although a more comprehensive genomic analysis of the *L. boulengeri* series, including nuclear genomes, is needed to shed light on which species are the ancestors of this curious lizard.

Although the CR is usually thought to be the fastest-evolving region of the mitogenome (Brown, George & Wilson, 1979), few reports explored the usefulness of the CR in phylogenetic analyses of lizards (Lalitha & Chandavar, 2018). Our study evidenced that this region may be highly informative for a variety of studies of the genetic variability of mitogenomes in lizards. For this purpose, a set of primer pairs were designed based on the conserved structures that flanked the repetitive sequences of the CR to reveal their variability (Table 4). We expect that the regions amplified by these primers pairs can provide very informative characters to analyze relationships of closely related species or at the infraspecific level and, to conduct population structure studies in *Liolaemus*. Moreover, since they were designed on highly conserved regions of the CR, they may be useful for other recently diverged lizard groups.

**Table 4:** Features of four primers to amplify the entire control regions of Iguanidae (primers CR) and tandem repeats of TAS domain of *Liolaemus* species (primers TAS).

Name	Sequence	Region to amplify
CR^fwd^	ARAGCRYYGGTCTTGTAARCC	trnT-trnF
CR^rev^	CKYGGCATYTTCAGTGCC	
TAS^fwd^	AGAGCATTGGTCTTGTAAGCC	trnT-TAS domain
TAS^rev^	TGGTYTCTCGTGAGATGGACG	

## Conclusions

We used low coverage whole genome sequencing of genomic DNA to assemble and annotate the first complete mitochondrial genomes from lizards of the Liolaemidae family, more precisely from three species of the genus *Liolaemus* (*L. parthenos*, *L. darwinii* and *L. millcayac*). The annotation of *Liolaemus* mitogenomes obtained in this study provides a comprehensive analysis of the nucleotide diversity of different regions for further development of useful genetic markers. Despite the high levels of sequence similarity among the three mitogenomes in most of their length, significant differences in copy numbers and motifs of the tandem repeats in the CRs were identified. These VNTRs arose as highly variable regions among *Liolaemus* species useful for future population and phylogenetic studies. Although a wide comparative genomic analysis is needed, the high similarity of the mitochondrial genomes evidences that the hypothesis that considers *L. darwinii* as a matrilineal ancestor of the hybrid triploid *L. parthenos* is plausible.

## Supplemental Information

10.7717/peerj.10677/supp-1 Supplemental Information 1 Putative secondary structures of the 22 tRNA genes of *L. darwinii* mitogenome. The tRNAs are represented by full names and IUPAC-IUB single letter amino acid codes. Anticodons are indicated between parentheses.

10.7717/peerj.10677/supp-2 Supplemental Information 2 Putative secondary structures of the 22 tRNA genes of *L. parthenos* mitogenome. The tRNAs are represented by full names and IUPAC-IUB single letter amino acid codes. Anticodons are indicated between parentheses.

10.7717/peerj.10677/supp-3 Supplemental Information 3 Putative secondary structures of the 22 tRNA genes of *L. millcayac* mitogenome. The tRNAs are represented by full names and IUPAC-IUB single letter amino acid codes. Anticodons are indicated between parentheses.

10.7717/peerj.10677/supp-4 Supplemental Information 4 Summary of sequencing and mitochondrial genome assembly outputs in *Liolaemus* species.

## Additional Information and Declarations

### Field Study Permissions

The following information was supplied relating to field study approvals (i.e., approving body and any reference numbers): The collection, euthanization, and preservation of specimens were carried out with the approval of Departamento de Fauna, Mendoza (permits 090/02, 034/06, 1397/07, 821/10, 296/10, 1259/10,1296/10).

### Data Availability

The following information was supplied regarding data availability: The mitochondrial genome sequences described here are accessible via GenBank: MT810467 (*L. darwinii*); MT810468 (*L. millcayac*); MT810469 (L. parthenos).
