# Supplementary figures and images for: Structure and comparative analysis of the mitochondrial genomes of Liolaemus lizards with different modes of reproduction and ploidy levels
Source: PeerJ. 2021 Mar 22;9:e10677. doi: 10.7717/peerj.10677 (PMC7996074; doi:10.7717/peerj.10677)

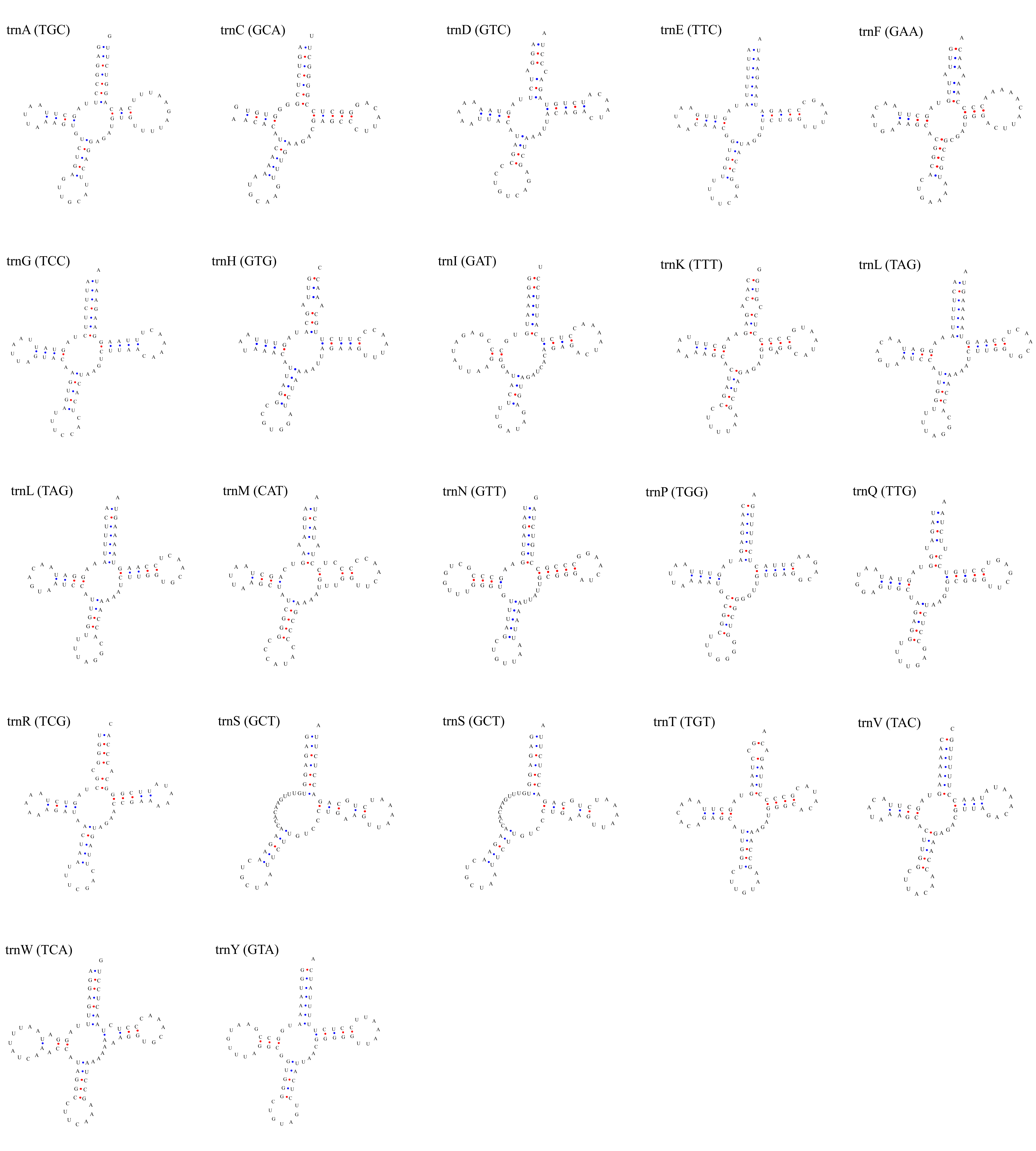

Supplement: Supplemental Information 1 — The tRNAs are represented by full names and IUPAC-IUB single letter amino acid codes. Anticodons are indicated between parentheses. [file peerj-09-10677-s001.png]

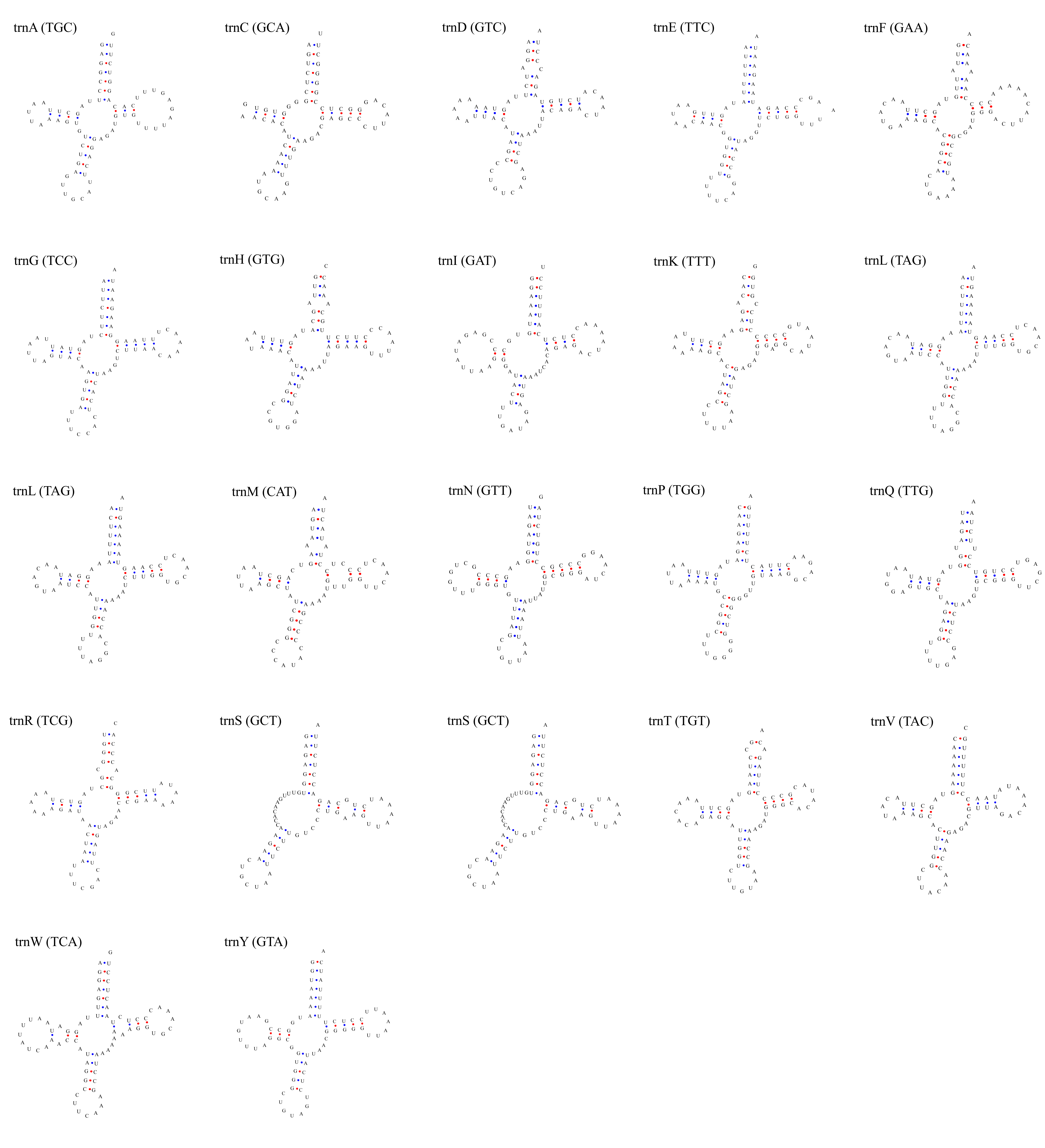

Supplement: Supplemental Information 2 — The tRNAs are represented by full names and IUPAC-IUB single letter amino acid codes. Anticodons are indicated between parentheses. [file peerj-09-10677-s002.png]

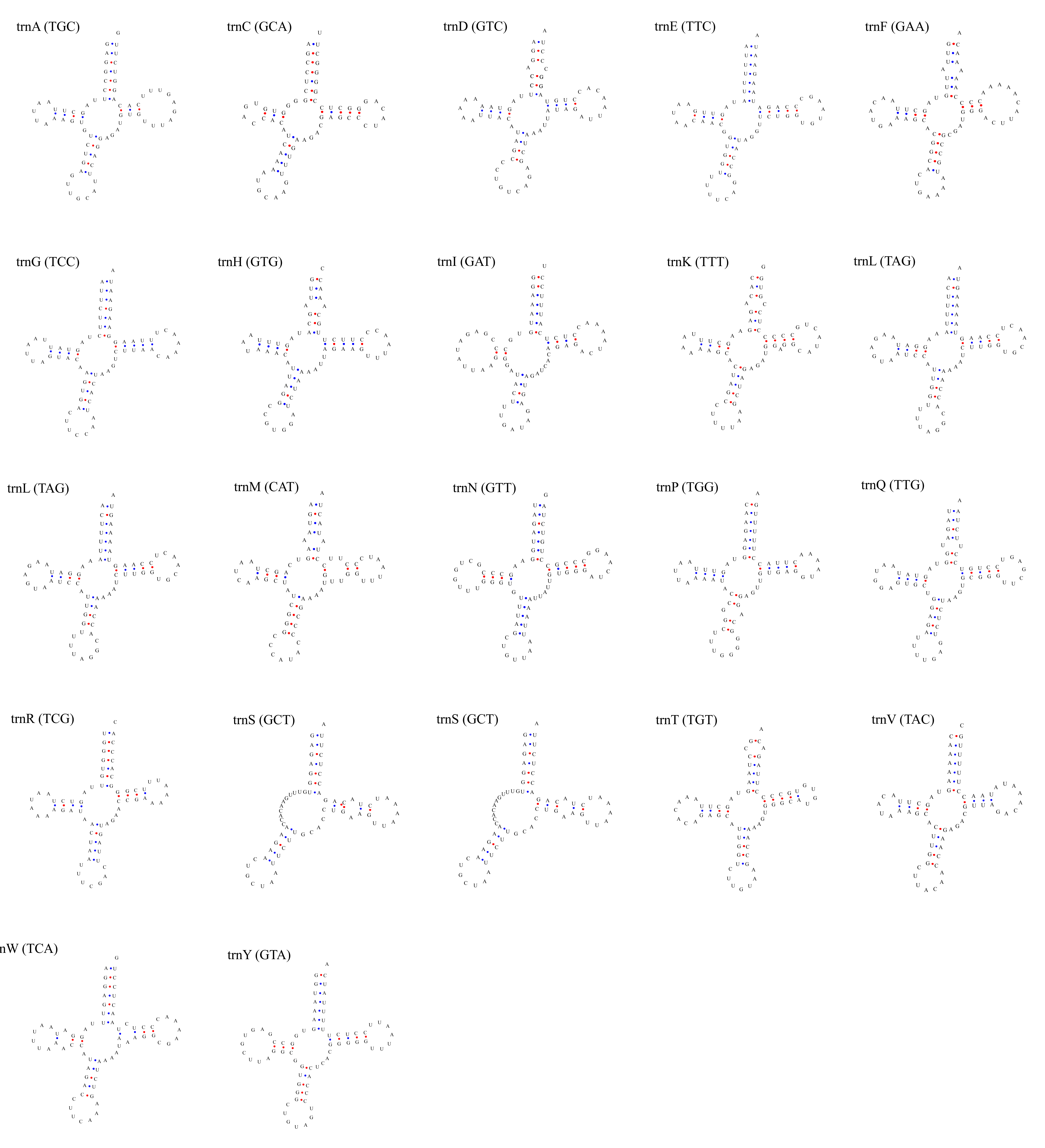

Supplement: Supplemental Information 3 — The tRNAs are represented by full names and IUPAC-IUB single letter amino acid codes. Anticodons are indicated between parentheses. [file peerj-09-10677-s003.png]
