# Supplementary material for: Structure and comparative analysis of the mitochondrial genomes of Liolaemus lizards with different modes of reproduction and ploidy levels
Source: PeerJ. 2021 Mar 22;9:e10677. doi: 10.7717/peerj.10677 (PMC7996074; doi:10.7717/peerj.10677)
Supplement: Supplemental Information 4 [file peerj-09-10677-s004.docx]

| Species | Total raw reads | Reads after quality filtering | Mitochondrial genome assembly | | | |
| --- | --- | --- | --- | --- | --- | --- |
|  |  |  | Aligned reads | Assembled reads | Average coverage | Assembled size (bp) |
| *L. darwinii* | 40,183,910 | 39,057,114 | 80,368 | 44,264 | 715 | 16,974 |
| *L. parthenos* | 43,295,362 | 42,135,996 | 130,578 | 58,026 | 1,171 | 16,838 |
| *L. millcayac* | 45,543,816 | 44,253,406 | 67,756 | 37,304 | 572 | 16,945 |
